# Supplementary material for: Real-Life Diagnostic Accuracy and Clinical Utility of Hepatitis B Virus (HBV) Nucleic Acid Testing Using the GeneXpert Point-of-Care Test System from Fresh Plasma and Dry Blood Spot Samples in The Gambia
Source: Microorganisms. 2024 Nov 9;12(11):2273. doi: 10.3390/microorganisms12112273 (PMC11596281; doi:10.3390/microorganisms12112273)
Supplement: Supplementary file 1 [file microorganisms-12-02273-s001.zip › microorganisms-3215920-supplementary figure S1.pptx]

## Slide 1
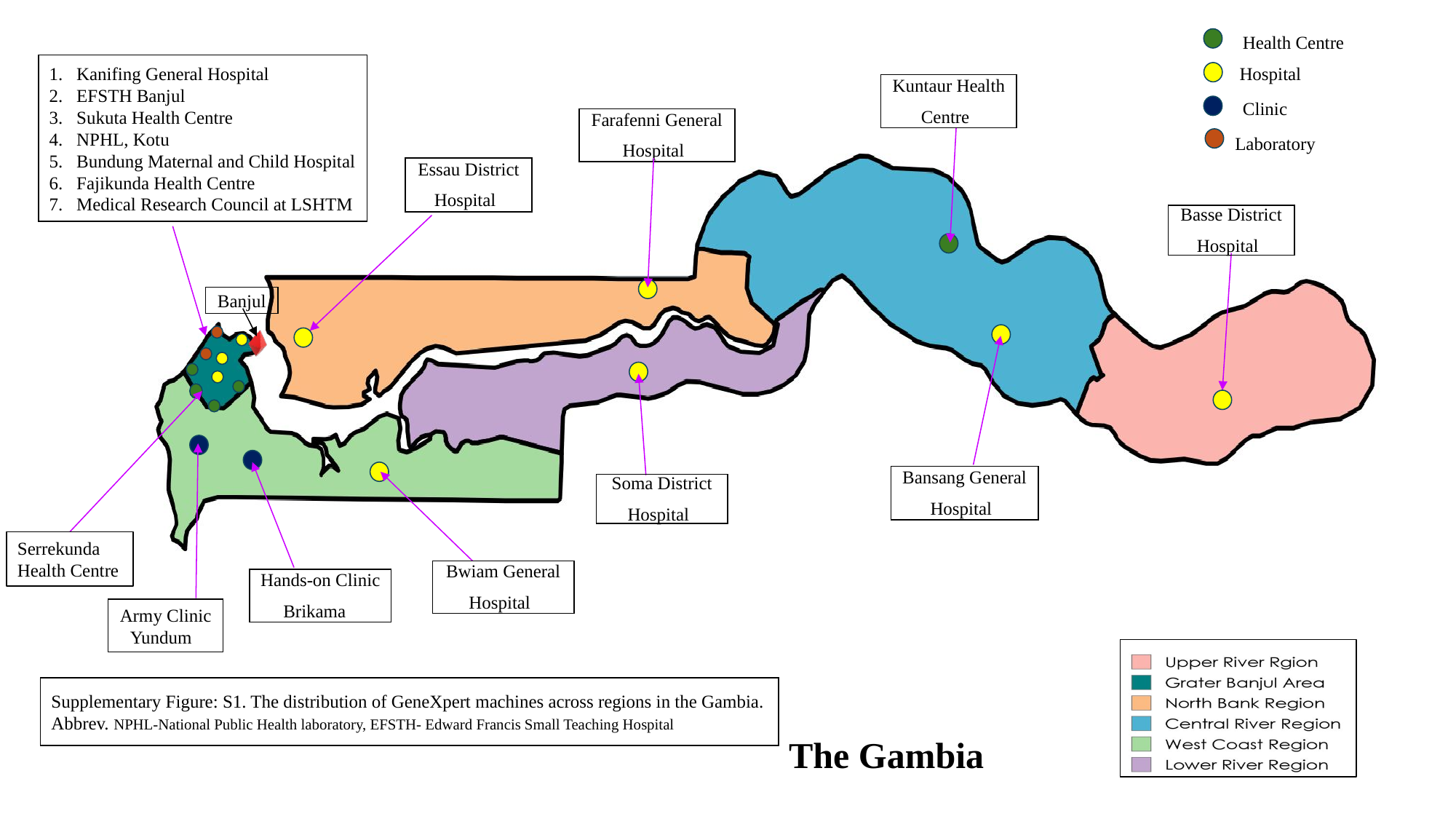

Health Centre
Kanifing General Hospital
EFSTH Banjul
Sukuta Health Centre
NPHL, Kotu
Bundung Maternal and Child Hospital
Fajikunda Health Centre
Medical Research Council at LSHTM
Hospital
Kuntaur Health Centre
Clinic
Farafenni General Hospital
Laboratory
Essau District Hospital
Basse District Hospital
Banjul
Bansang General Hospital
Soma District Hospital
Serrekunda Health Centre
Bwiam General Hospital
Hands-on Clinic Brikama
Army Clinic Yundum
Supplementary Figure: S1. The distribution of GeneXpert machines across regions in the Gambia.
Abbrev. NPHL-National Public Health laboratory, EFSTH- Edward Francis Small Teaching Hospital
The Gambia
